# Consistent differences in a virtual world model of ape societies

**DOI:** 10.1038/s41598-020-70955-6

**Published:** 2020-08-21

**Authors:** Bart J. Wilson, Sarah F. Brosnan, Elizabeth V. Lonsdorf, Crickette M. Sanz

**Affiliations:** 1 Economic Science Institute & Smith Institute for Political Economy and Philosophy, Chapman University, Orange, CA USA; 2 Departments of Psychology and Philosophy, Neuroscience Institute, Language Research Center and Center for Behavioral Neuroscience, Georgia State University, Atlanta, GA USA; 3 Department of Psychology, Biological Foundations of Behavior Program, Franklin and Marshall College, Lancaster, PA USA; 4 Department of Anthropology, Washington University, Saint Louis, MO USA; 5 Congo Program, Wildlife Conservation Society, Brazzaville, Republic of the Congo

**Keywords:** Social evolution, Animal behaviour, Behavioural ecology

## Abstract

Practical and ethical constraints limit our ability to experimentally test socioecological theory in wild primates. We took an alternate approach to model this, allowing groups of humans to interact in a virtual world in which they had to find food and interact with both ingroup and outgroup avatars to earn rewards. We altered ratios and distributions of high- and low-value foods to test the hypothesis that hominoids vary with regards to social cohesion and intergroup tolerance due to their feeding ecology. We found larger nesting clusters and decreased attacks on outgroup competitors in the *Bonobo* condition versus the *Chimpanzee* condition, suggesting a significant effect of feeding competition alone on social structure. We also demonstrate that virtual worlds are a robust mechanism for testing hypotheses that are impossible to study in the wild.

## Introduction

In recent years, computational models and simulations have emerged as complementary tools to more traditional studies of primate behavior in the naturally occurring world. While technological advances have enhanced observers’ abilities to study complex behaviors (such as collective movement^1^) and enabled more detailed tracking of individual animals^2^, such approaches nonetheless remain expensive, difficult, and unethical in some species and contexts, and tracking natural movements does not allow for experimental hypothesis testing. While other fields, such as experimental archaeology have long enlisted humans to replicate tasks of other species to gain insights into evolutionary processes, experimental paradigms introducing human avatars to virtual primate habitats have yet to be explored. These alternate worlds offer the possibility of modeling key features and measuring responses to those features that are impossible to manipulate in natural conditions. One area in which such approaches and hypothesis testing would be particularly useful is in understanding the different social structures in the genus *Pan*, chimpanzees and bonobos^3^.

Chimpanzees and bonobos shared a common ancestor 1–2 million years ago^4^, with increasing evidence of more recent contact and admixture^5^. Despite being very closely related and exhibiting the same general fission–fusion grouping pattern (wherein temporary subgroups of different community members can change throughout the day), some aspects of their sociality differ dramatically. In particular, even taking into account differences across subspecies and populations of chimpanzees, bonobos show far greater intergroup tolerance^3,6–8^. Male chimpanzees routinely patrol the boundaries of their territories for opportunities to expel intruders and/or lone members of other groups^9–11^ and most long-term study sites have reported killing of individuals in other groups^12–15^. Bonobos show milder aggression during intergroup encounters^9,16,17^, and there are no documented cases of intergroup killing^3^. They may even show affiliative interactions with members of other groups during intergroup encounters^18–21^, including food sharing^22^ and facilitating outgroup member access to food^23^. Within groups, these species also exhibit different patterns of gregariousness. Chimpanzee males are typically more gregarious than females and are consistently dominant to them, whereas female bonobos are more likely to be part of a larger party and are dominant to males^3,24^. While it is tempting to ascribe polarity of change for phenotypic changes, we refrain from characterizing such evolutionary relationships as they are difficult to resolve when the state of the last common ancestor is unknown^25,26^. One hypothesis suggests that these differences emerged due to differences in their feeding ecology that selected for reduced aggression (i.e. the self-domestication hypothesis^27^). While there is substantial variation in the feeding ecology of *Pan*, and bonobos reside within the range of habitats seen across chimpanzee subspecies^3^, there are broad differences. Chimpanzees generally favor ripe fruits, which tend to be temporally and spatially patchier than leaves or herbs^22,23^. In contrast to chimpanzees’ sustained frugivory, bonobos consume a relatively high proportion of lower-quality resources, including leaves and terrestrial herbaceous vegetation (THV:^28–30^) throughout the year. Therefore, absent other factors, chimpanzees’ greater reliance on a valuable, clumped resource should lead to (1) greater between-group contest competition in chimpanzees, who must defend fruit from outsiders, than bonobos and (2) greater within-group cohesion in bonobos, who are not competing with one another for THV, than in chimpanzees.

In this study, we report the results of an immersive virtual world experiment that tested the impact of different food values, availability, and distribution on social behavior. In virtual environments, we can control both environmental factors and the types of possible interactions to determine how changing conditions influence decision-making. While commonly used in experimental economics^31–39^, virtual environments are a relatively new and flexible approach for testing hypotheses about the influence of ecology on behavior. We hypothesized that the distribution, availability and value of food is a key element in determining both within-group social cohesion and the nature of interactions with outgroup members in apes. We tested this using a model system in which humans controlled virtual hominoids who interacted with one another in real time across a landscape in which we systematically varied food resources designed to emulate key aspects of *Pan* ecology. Specifically, we predicted that when total food was held constant, humans in a world with high value foods that were patchily distributed in space and time would form smaller nesting groups (our proxy for group cohesion) and would have higher rates of aggression towards a non-group member (our proxy for intergroup aggression). In contrast, humans in a world with lower value foods that were distributed more evenly in space and time would form larger nesting groups and show lower rates of aggression towards a non-group member.

## Results

A total of 96 subjects in 8 12-person sessions, split across two treatments, interacted as avatars in 35 90-s periods (representing days; 75 s of day (including 5 s of dusk) and 15 s of night). Their goal was to earn as many points as possible, which were converted into US Dollars (at a 1:1 ratio) at the end of the experiment. Avatars were numbered and color coded so that individuals could identify one another. During the day, avatars could earn points that were directly converted into cash earnings by foraging for one of two types of food (“fruit” and “grass”; see below for details) and participating in a generic social interaction that was a proxy for beneficial social engagement. Fruit was high value but replenished slowly (never within the same day), and was always scarce, whereas grass was low value but infinitely renewable, that is, it was continuously available at the site it appeared at each day. The social interaction was labeled “health” for the participants but hereafter we refer to it as “grooming”, for it represents all directional social interactions that provide a direct benefit to one other avatar at a time. Because grooming was equally important to earning points in both conditions, it was not useful for measuring differences in sociality between the two. At night, remaining stationary in nests (all extant apes exhibit such nesting behavior) increased points. (See the supplementary online material for our precise language. For example, we did not use the words *grooming, chimpanzee*, or *bonobo*.)

In both conditions, the world was a rectangle with two “groves” of trees, one in the north and one in the south, which was designed to make it costly for avatars to congregate around a single supply of fruit, as apes in the naturally occurring world must search out fruit from dispersed groves. The amount of fruit was equally distributed between northern and southern trees, and grass was randomly distributed throughout the world so that there was no caloric incentive to prefer one area of the world over another. Fruit trees remained in the same location, but flowered and bore fruit in a cyclical pattern. Fruit was thus not available on each tree each period, but avatars could predict that it would be available in a day or two based on the flowering. Moreover, once a fruit was eaten in a given period, it was no longer available. Avatars could not guard fruit or exclude others from a tree. The location of grass changed each day as well, so subjects could not obtain enough food without moving, but within a day the grass continuously renewed and multiple individuals could feed on the same patch at the same time. The aggregate amount of food was held constant between *Chimpanzee* and *Bonobo* conditions. There was three times as much fruit per day (120 vs 40 pieces) in the *Chimpanzee* treatment vs the *Bonobo* treatment, but it took three times as long to forage on grass in the *Chimpanzee* treatment. Note that this was not meant to reflect naturally occurring handling times, but provided a way to incentivize different food choices while keeping the rate of food consumption the same across conditions.

Randomizing the location of the grass around the world and having trees fruit at different times made the problem of forming and maintaining groups nontrivial. In other words, before conducting the experiment we did not know if our design choices would induce any grouping behavior. The virtual environment was sufficiently large relative to avatar speed that it took 22% of the day to walk between the two groves of trees. Consistent with foraging in a forested environment, subjects could not see the entire world, but only a limited range around them. A map in the upper left corner of the screen displayed their location as well as the location of the trees (but not whether they were fruiting), which was designed to be a proxy of the mental maps apes have of their environment^40^. Subjects could call to one another over a greater distance and tell from what direction others’ calls emanated.

Finally, subjects, at a severe potential cost to themselves, could also individually attack a lone outsider, explicitly termed a “pirate”, who ate the fruit, but not less valuable grass. If one avatar attacked the pirate, the avatar incurred a significant cost and the pirate continued eating fruit. Subsequently, any avatar within the viewing window received a message indicating the outcome of two simultaneous attacks. If two avatars attacked the pirate, neither incurred a cost, and the outsider would leave for the rest of the day only to return the next day. Likewise, nearby avatars then received a message explaining three simultaneous attacks: if three or more group members attacked the pirate, it was “killed” and did not return in future days, although unannounced to the participants, there were a total of three pirates in each world; if all three were killed, no additional pirates appeared. Note that we intentionally made a solo attack extremely costly because solo attacks are not reported in the wild. However, we did not disallow solo attacks because one of our goals was to see whether such behavior emerged endogenously. In addition, this approach required minimal instruction and no explicit rules restricting behavior. This latter point was extremely important, as our goal was to see how people would explore the space and what decisions they would make *without* instruction, which could bias their subsequent decisions. An online video (https://www.youtube.com/watch?v=i0o_9nf2wwc) illustrates the subjects’ tasks in the virtual world and provides the experimental context.

**Figure 1:**
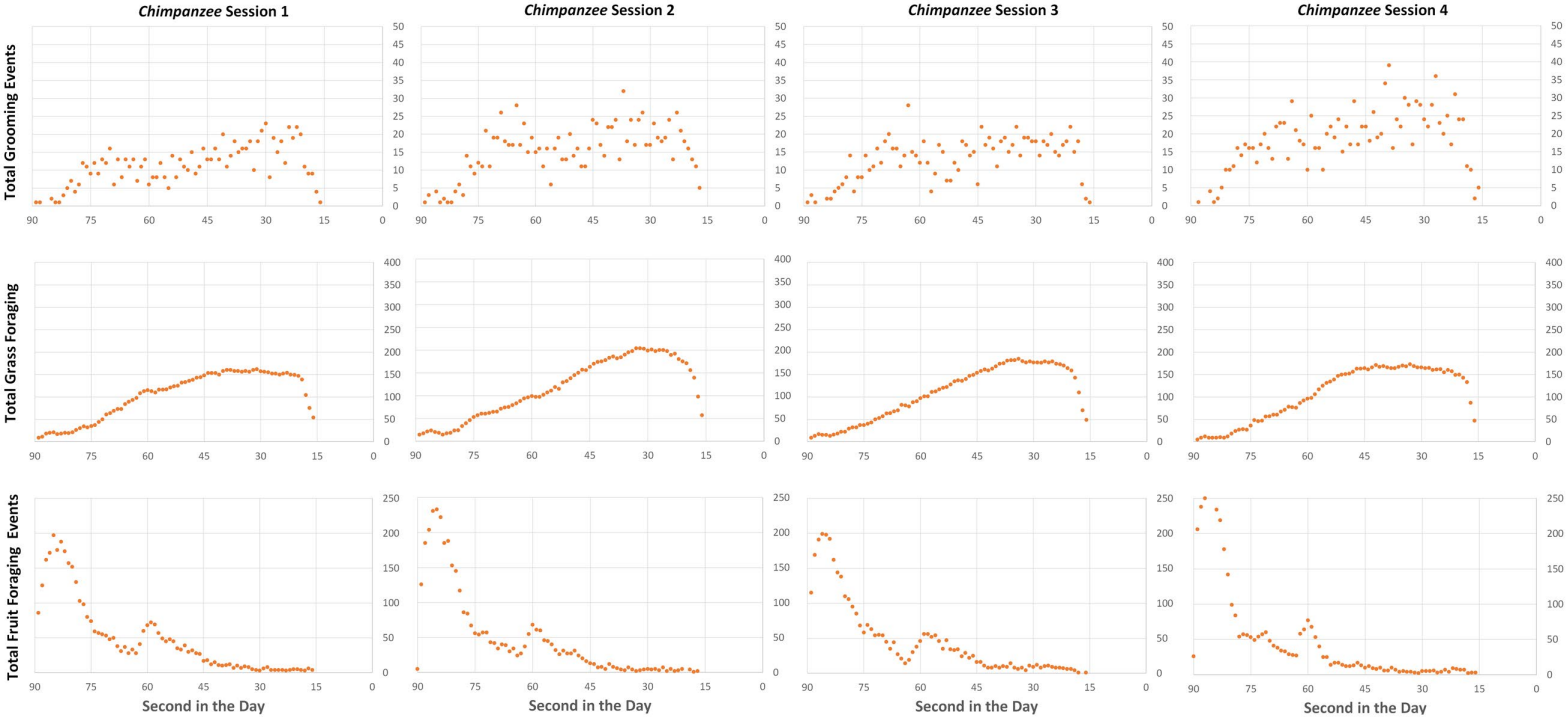
Grooming and foraging over the course of the day in the *Chimpanzee* treatment, summed over 35 days.

**Figure 2:**
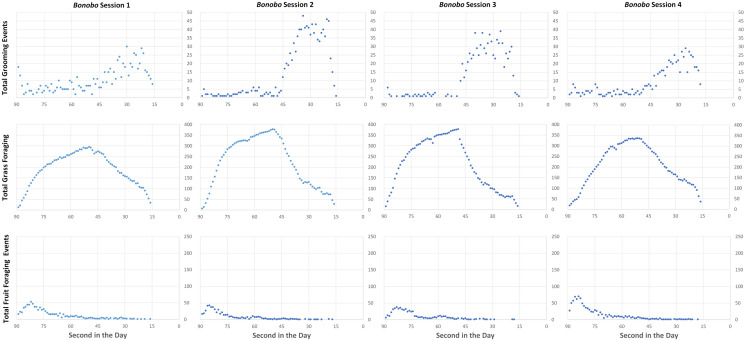
Grooming and foraging over the course of the day in the *Bonobo* treatment, summed over 35 days.

Given that events during the day occurred in real time at the discretion of the participants, and may depend on idiosyncratic social temperaments, a daily pattern of the events was ex ante unpredictable. Our first result establishes the consistency of behaviors across four different sessions of a treatment in response to the biological imperatives we induced in the experiment. In Figs. 1 and 2, we report the total number of grooming, grass foraging, and fruit foraging events over the course of a day (summed over all 35 days) for each session in the *Chimpanzee* and *Bonobo* treatments, respectively.

### Global differences

The results in Figs. 1 and 2 indicate a consistency with which the sessions replicated a daily pattern in the two different ecological environments. Such consistency in an experiment with a relatively unstructured decision space indicates that we have created an environment in which the participants responded to the incentives we presented. In other words, we appear to have designed an experiment such that rewards of the experiment (the money they earned for their choices) were high enough to maintain the attention of the participants, i.e., “the reward structure dominates any subjective costs (or values) associated with participation in the activities of an experiment” (^41^, p. 934).

One key design goal of our virtual environment was that the virtual worlds contained the same amount of total food even though the treatment conditions varied the amount of the fruit and processing time for grass. This goal was achieved; the average, maximum, and minimum earnings for all participants were very similar for the *Bonobo* and *Chimpanzee* treatments—respectively, US$15.98 (s.d. = $9.23) vs. US$16.29 (s.d. = $8.00), US$27.87 vs. $27.53, US$3.00 vs. US$2.85—indicating that the environments, by design, were indeed equally challenging for the participants. There was no significant difference in average session earnings (Mann–Whitney *U*_4,4_ = 8 > critical value = 0, *α* = 0.05, two-tailed test). Nonetheless, we observed differences between the treatments (see Figs. 1 and 2). The hominoids in the *Chimpanzee* treatment spent the earliest part of the day (15 s) foraging for fruit, followed by a slow sustained increase in grass foraging and a variable, but a flat rate of grooming. In the *Bonobo* treatment, hominoids quickly increased their grass foraging over the first half of the day (40 s) and then spent the rest of the daylight time (35 s) grooming. Consistent with the different ecological inducements, *Bonobo* hominoids spent very little time foraging for fruit as compared to their *Chimpanzee* counterparts, and *Chimpanzee* hominoids spent much less time foraging for grass. While there were subtle differences in the patterns of daily events within a treatment (some social groups groomed more than others as compared to other sessions in the same treatment condition), the data in Figs. 1 and 2 visually indicate that *Chimpanzee* sessions were more similar to each other than they were to *Bonobo* sessions and vice versa.

**Figure 3:**
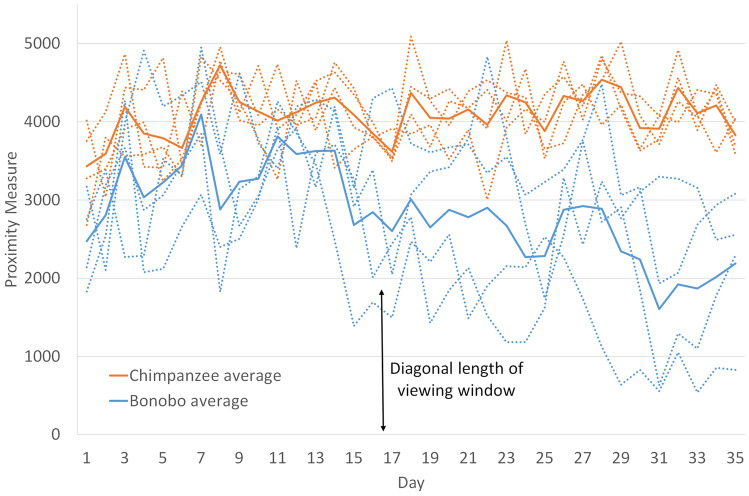
Nesting proximity by day and treatment.

The nesting locations of the avatars indicated with whom the avatars concluded their day’s activities and with whom they began the next day; this was our measure of social affiliation since it earned no points for social partners (like grooming did) and was therefore a measure of subjects’ endogenous affiliation choices. If all 12 avatars decided to nest, there were 12*C*2 = 66 combinations of unique distances between the avatars. To quantify the avatars’ proximity to one another at the end of a day, we summed the unique distances between all avatars who chose to nest. As some avatars occasionally decided not to nest (and instead stood in place or walked around), we divided the sum by the actual number of nest combinations for that day to ensure the distance measure was comparable across days. (For example, if only 10 avatars nested in a day, there are only 10*C*2 = 45 distances between 10 avatars that day). Figure 3 illustrates the nesting proximity of avatars by day, with sessions represented by dashed lines and treatment averages across all sessions represented by solid lines (orange for *Chimpanzee*, blue for *Bonobo*). Lower numbers indicate closer nesting proximity within the session. The trendline for the *Bonobo* average is decreasing (− 40.6 pixels/day) at a statistically significant rate (*p*-value < 0.0000). The trendline for the *Chimpanzee* average is slightly increasing (8.4 pixels/day) but is statistically insignificant (*p*-value = 0.0770).

Changing the distribution, availability, and processing time of food resulted in distinct patterns of association in these equally challenging environments. For the first third of a session, the hominoids in the two treatment conditions were roughly equally close to (or evenly dispersed from) each other in the virtual world. Beginning on day 15, the *Bonobo* hominoids began to associate in one larger group of 9 to 12 avatars while the *Chimpanzee* hominoids separated into northern and southern subgroups of 3 to 6 avatars (a group was considered any avatars who were in same overlapping range of vision). The average measure of proximity by session for all 35 days was significantly lower in *Bonobos* than *Chimpanzees*, (Fig. 3: Mann–Whitney *U*_4,4_ = 0, *p*-value = 0.05, two-tailed test). Because the amount of fruit was equally distributed between the northern and southern trees, splitting into two subgroups was the most efficient way for hominoid avatars to forage in the *Chimpanzee* treatment. The low variance of proximity in the *Chimpanzee* sessions indicated that half of the avatars spend their days and nights by the north trees and the other half by the southern trees.

In the two *Bonobo* sessions that showed the closest proximity in nesting by the end of the experiment, 83% to 100% of the avatars gathered to nest as a single group within the same viewable area (in the wild, bonobos form larger nesting aggregations^42^ and generally show closer proximity than chimpanzees^43^). When the patch of grass randomly moved the next day, this large group moved together to find a new patch. Such collective movement among avatars was surprising and unexpected given our incentive structure. Further, large aggregations are not necessary for the *Bonobo* hominoids to successfully feed themselves and socialize. Two or even three subgroups spread out over the virtual world would be sufficient to maximize one’s intake of grass and to be maximally groomed each day (for details on the benefits of grooming, see the Methods/Experimental Design section below). However, presumably because they do not need to compete for limited fruit, the avatars in the *Bonobo* condition tend to form one large group visible to everyone. As soon as they were satiated on grass, they continuously groomed each other, which is the only overt prosocial interaction that could be expressed in the world.

The other two *Bonobo* sessions also have stable cohesive groups of 6 and 9 avatars, which, by the end of the session, spend their entire days together. In the former session, the remaining 6 avatars form stable groups of 4 and 2 avatars. In the latter session, the remaining 3 avatars tend to wander about on other own in the same neighborhood, but they do not stay in continuous view of each other like the 11-, 12-, 6-, and 9-avatar groups above do.

### Aggressive behavior

**Figure 4:**
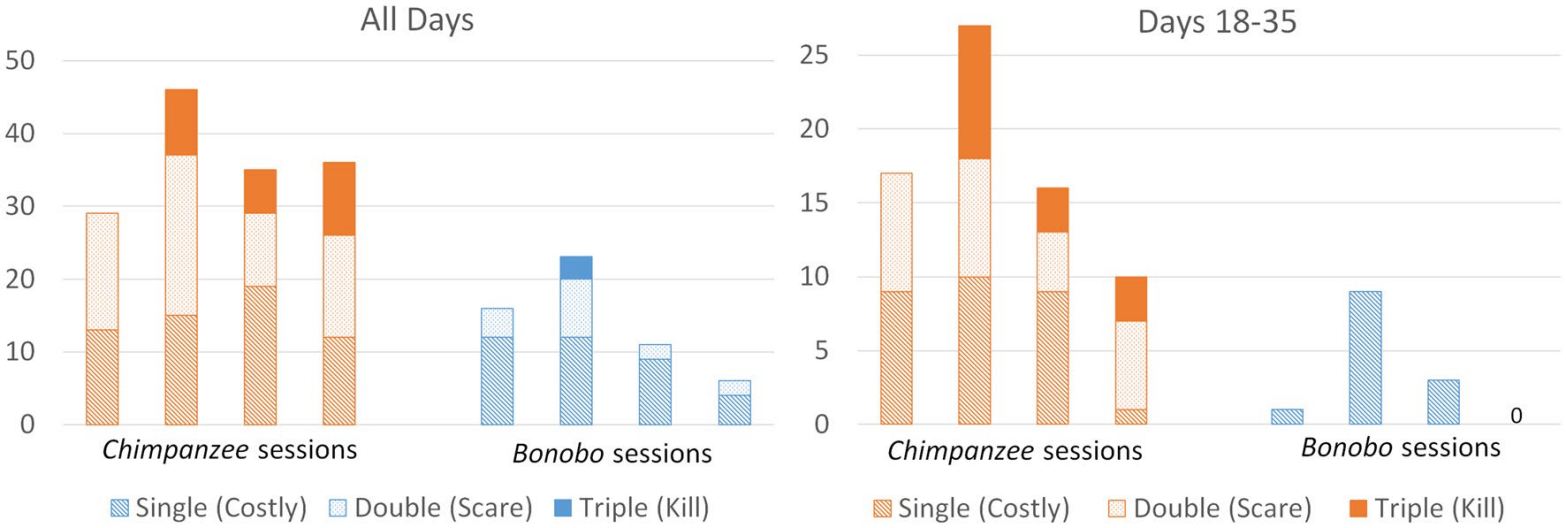
Single and coalitionary attacks on outgroup members by session. *Note* The height of the bar indicates the total number of attacks on the pirate by session for all days (left) and the last half of the session (right).

Having established consistent foraging and grooming behaviors within both treatment conditions and having found distinct social grouping behaviors between the two treatment conditions, our final analysis tested whether avatars in the two treatments would respond differently to outgroup members. A single pirate would appear each period unless a total of three pirates had been killed, in which case pirates never appeared again for the remainder of the session. Indeed, *Chimpanzee* hominoids more frequently attacked the pirate avatar than *Bonobo* hominoids did. Figure 4 reports the number of single attacks, attacks as pairs, and attacks as triplets on the pirate avatar.

Hominoids in both treatment conditions attacked the outgroup pirate at the beginning of the session, but they rarely killed it with three attackers. *Chimpanzee* hominoids continued to attack the pirate throughout the second half of the session (76 total attacks in the first half and 70 attacks in the second half), and in three of the four sessions they killed at least one pirate with three joint attacks. Avatars in two of the *Chimpanzee* sessions killed all three pirates, permanently ridding themselves of the rival foragers. *Bonobo* hominoids, on the other hand, substantially reduced their attacks (43 total attacks in the first half and only 13 in the second half) and *never* jointly attacked the pirate in the second half of the session. There were no attacks in the fourth *Bonobo* session for the last half of the session. Using the total number of attacks by session for all 35 days, *Chimpanzees* attack significantly more frequently than *Bonobos* (Mann–Whitney *U*_4,4_ = 0, *p*-value = 0.05, two-tailed test).

These results demonstrate that differences in the value and distribution of food can drive differences in both within-group cohesion and out-group behavior, such that when food abundance is held constant, virtual hominids in an environment in which high-value food is centralized in ephemeral clumps form smaller social groups and attack outgroup members more than virtual hominids in an environment with widely distributed low-value food. Of course, model systems by design highlight specific features for hypothesis testing, but these results suggest that this difference could have led to the actual differences observed between chimpanzee and bonobo social structure. While ecology is not the only difference between the species, our results support one widely accepted hypothesis suggesting that the initial divergence was based on geographic separation rather than behavioral differentiation^3^, and that these ecological differences then led to the differing social patterns. Indeed, it is reasonable to hypothesize that this became self-reinforcing, with less need to defend food resources and increased opportunities for group cohesion working in tandem to select for decreased aggression and increased social tolerance (i.e.^27^). Moreover, the coherence and consistency of avatar behaviors across sessions, with very minimal instruction to the participants or experimental constraint, suggests that these virtual worlds are a robust method for testing questions about the role of ecology and, potentially, social structure (i.e., by changing the costs and benefits of social interaction) on primate decision-making. In particular, this method may allow for experiments that are impossible or unethical to conduct in wild primates and difficult to model due to unknown parameters, thereby providing new opportunities for advancing our understanding of the evolution of primate sociality.

## Methods/experimental design

The experimental design directly tests the hypothesis that the distribution of food sources could shape social structuring in apes. Specifically, we predicted that clumped, scarce, high-value food sources, like fruit, would induce relatively small groups of apes with high aggression, including lethal violence, towards outsiders, whereas plentiful and smoothly distributed, albeit lower-value, food sources, like terrestrial herbaceous vegetation, would induce relatively large groups of apes with little to no aggression towards outsiders. Our hominoid subjects, undergraduate students at Chapman University, lived in a virtual environment for nearly an hour where they could forage for food (“fruit” and “grass”) and “groom” with one another for points which were convertible into cash (subjects earned, on average $16.13 for participating). Subjects, at a severe potential cost to themselves, could also attack a lone (simulated) outsider (the “pirate”) who ate fruit but not grass. An online video (https://www.youtube.com/watch?v=i0o_9nf2wwc) illustrates the subjects’ tasks in the virtual world and provides the experimental context. The full instructions from the experiment are included in the Supplemental Online Methods. We received approval from Chapman University’s IRB to conduct the experiment, and all methods were performed in accordance with the relevant guidelines and regulations. We also received informed consent from all participants prior to their participation as a human subject in this social research.

**Figure 5:**
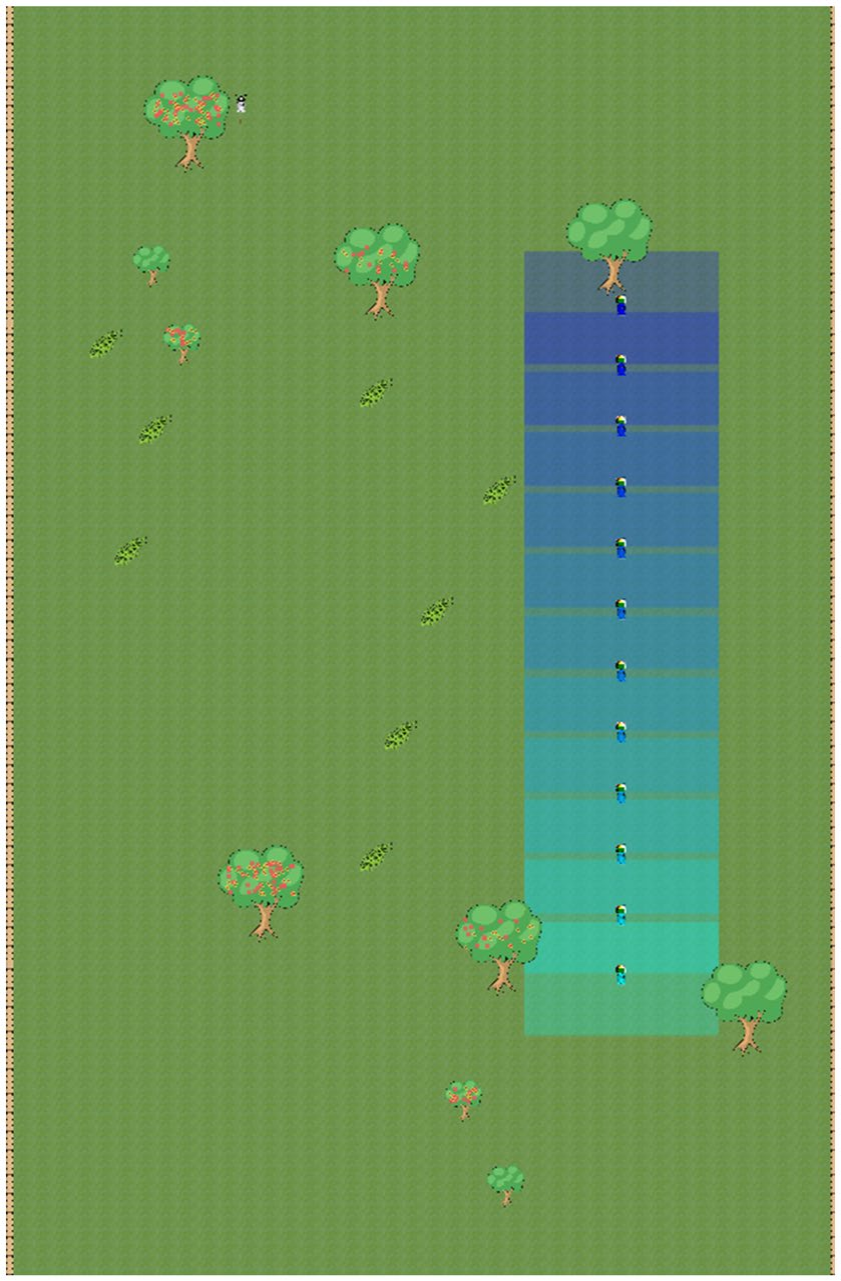
Bird’s eye view of the virtual world with the avatars in their starting positions.

In each session, we randomly manipulated the ecology for a twelve-person group of subjects (6 men and 6 women) seated at visually isolated computer carrels. We conducted four independent sessions for each of the two treatment conditions. In the first treatment condition, *Chimpanzee*, 120 pieces of fruit were distributed across 3 of 5 trees in the North and 3 of 5 trees in the South. Figure 5 displays a bird’s eye view of the virtual world. The fruit was equally divided between the Northern and Southern trees. A different set of 3 trees bore the same total amount of fruit each day. Flowers on the trees indicated on which tree and how much fruit would be available the next day.

We measured calorie intake in the experiment as the total number of pieces (or units) of food, where one piece of fruit equals one unit of food. To reflect metabolism of food, the avatars lost (“digested”) up to 11 units of food each day at two discrete times of the day (4 in the middle of the day and 7 before dawn), which meant that fruit was always scarce for the apes (120 < 12 × 11 = 132). If an avatar did not have 4 or 7 units of food to lose, they lost whatever stock they currently had. Grass was also available for consumption and was never scarce, but yielded fewer units such that there was not enough time in the day to replace 11 units of digested food. Specifically, it took 10 times as long to graze the equivalent of one piece of fruit. There were eight patches of grass randomly distributed around the world each day.

In the second treatment condition, *Bonobo*, 40 pieces of fruit were distributed across 3 of 5 trees in the North and 3 of 5 trees in the South. The avatars similarly digested food twice per day, but grass, which was never scarce, was nutritious in the sense that there was plenty of time in the day to replace the 11 units of food. It only took 3.33 times as long to graze the equivalent of one piece of fruit. Thus, there was three times as much fruit available in the *Chimpanzee* treatment as compared to the *Bonobo* treatment, but it took three times as long to graze grass in the *Chimpanzee* treatment. Such compensating differences meant that there was roughly the same aggregate amount of food available in both treatment conditions.

Everything else was held constant across the two treatment conditions. The experiment induced sociality by making grooming (a catch-all, directional social interaction) necessary for accumulating cash-earning points. An avatar could groom another avatar once every four seconds but could not gain these units by grooming itself. The amount of grooming received was capped at 10 units. These units, however, slowly depleted as the avatar walked around the world. Walking was thus costly as the units did not deplete if the avatar remained stationary. Below 4 units, the points that the avatar has accumulated for earnings began to fall each second. Above 7 units, the points for earnings increased each second. Between 4 and 7 units of grooming, there was no change to the points for generating earnings. Every individual was thus induced to remain in the company other avatars. The food units worked the same way: they were capped at 10 units with thresholds of 4 and 7 units for decreasing and increasing cash earning points, respectively.

A day in the virtual world lasted 90 s and was repeated 35 times. Daylight lasted for 75 s each day, and the light began to set at 20 s remaining. The subjects could choose to nest for the last 15 s of the day. The points for earning cash slowly increased while an avatar nested (0.3 points per second). Avatars who continued to walk around at night could not forage or groom and did not rejuvenate their cash earning points. As it was our only voluntary measure of social proximity, since it was not explicitly rewarded, we used the nesting location data to create a measure of the proximity of a group and to test our hypothesis on the ecological influences of grouping behavior in apes. Avatars could not nest during daylight.

The virtual world was large relative to the range within which avatars could view food and each other. It took approximately 20 s of valuable time (22 percent of the day) to move from the southernmost tree in the North to the northernmost tree in the South. More specifically, the size of the virtual world was 6,720 × 10,500 pixels, but the viewing window for an individual avatar was only 1584 × 947 pixels. (The blue rectangles in Fig. 5 display the viewing windows.) Avatars could communicate with each other by calling to each other. All avatars within a radius of 2,200 pixels could see the image of a sound wave on their screen in the direction from which an avatar was calling to them (see Supplementary Materials for details). The image contained no further information as to the content/reason for the call. To make the individual avatars identifiable to each other, the individual avatars were numbered from 1 to 12 and color-coded dark blue to teal.

An outgroup avatar, explicitly called a pirate in the instructions, randomly appeared once every day at either the largest fruit-bearing tree in the north or the largest fruit-bearing tree in the south. The pirate was colored gray and sported a pirate cap to distinguish it as an outsider to the group of blue-shaded avatars. The pirate was a computer simulation who ate one piece of fruit every 4 s on average. Avatars could walk up to the pirate and attack it. Once attacked, a beam linked the avatar and interloper for the next 10 s. If no other avatar attacked the pirate during those 10 s, the avatar lost 10 of their cash-earning points, which was 10% of the maximum 100 points possible. To incur such a potential cost, an avatar must have had at least 10 points to attack a pirate. If no other avatar attacked the pirate within 10 s, the following note appeared above the pirate for all in viewing range to read for approximately 17 s: “Pirates will be scared away if 2 avatars attack it simultaneously, and you will incur no damage.” Only if an avatar attacked a pirate did the participants in the viewing range receive such information. If two avatars attacked and simultaneously linked themselves to the pirate, a different note appeared: “3 simultaneous attackers will kill this pirate, and you will incur no damage.” A dead pirate remained visible until night came. The first pirate was identified as “1 of 3 pirates.” Pirates stopped appearing once a group killed the third pirate. We used the number of single, double, and triple attacks on pirates to test our hypothesis on the ecological influences of intergroup aggression in *Pan*.

Such explicit language and images of a pirate were intended to explicitly draw attention to the avatar as an interloper so as to create a robust test of the hypothesis that different ecologies differentially induce aggression toward outsiders. Before conducting the experiment, we did not know if such a design choice would induce rampant attacks or few attacks in either or both treatment conditions. Such a stark context provided a clear baseline against which to assess our design choices in the event our results failed to differentiate by treatment condition.

## Supplementary information

Supplementary information.

## Data Availability

The data that support the findings of this study are archived at Chapman University Digital Commons (10.36837/chapman.000180).
