# Supplementary material for: Consistent differences in a virtual world model of ape societies
Source: Sci Rep. 2020 Aug 21;10:14075. doi: 10.1038/s41598-020-70955-6 (PMC7442632; doi:10.1038/s41598-020-70955-6)
Supplement: Supplementary file 1 — Supplementary information. [file 41598_2020_70955_MOESM1_ESM.docx]

Supplementary Materials for

Consistent Differences in a Virtual World Model of Ape Societies

Bart J. Wilson, Sarah F. Brosnan, Elizabeth V. Lonsdorf, and Crickette M. Sanz

Correspondence to: bartwilson@gmail.com

**This PDF file includes:**

Figure of the Stock of Food and Health (Grooming), Flow of Wellness, and Total Earnings for Participants as Displayed for the Participants

Table of Parameter Details

Materials and Methods

Figure of the Stock of Food and Health (Grooming), Flow of Wellness, and Total Earnings as Displayed for the Participants


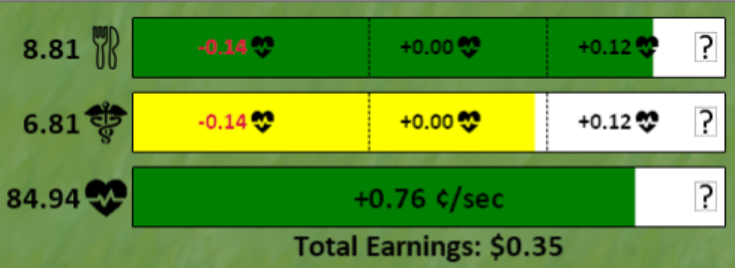


Table of Parameter Details

|  | *Chimpanzee* Treatment | *Bonobo* Treatment |
| --- | --- | --- |
| Number of Participants per Session | 12 | 12 |
| Number of Days | 35 | 35 |
| Length of Day (including Night) in Seconds | 90 | 90 |
| Length of Night in Seconds | 15 | 15 |
| Starting Wellness Points | 75 | 75 |
| Fruit Foraging Rate Maximum | 1 unit/second | 1 unit/second |
| Healing Rate Maximum | 1 unit/4 seconds | 1/4 seconds |
| Increase of Wellness per Second if Nesting at Night | 0.3 | 0.3 |
| Conversion of Wellness in Earnings (Cents) | 0.9 | 0.9 |
| Maximum Stock of Food | 10 | 10 |
| Minimum Stock of Food to Add Wellness Points | 7 | 7 |
| Wellness Points Added per Second with Minimum Stock of Food to Add Wellness Points | 0.12 | 0.12 |
| Minimum Stock of Food to Not Lose Wellness Points | 4 | 4 |
| Wellness Points Lost per Second if Stock of Food is Less Than Minimum Stock of Food to Lose Wellness Points | 0.14 | 0.14 |
| Food Stock Lost Midday (38 Seconds into Day) | 4 | 4 |
| Food Stock Lost at Night (75 Seconds into Day) | 7 | 7 |
| Food Stock Lost per Second of Walking | 0.11 | 0.11 |
| Maximum Stock of Health | 10 | 10 |
| Minimum Stock of Health to Add Wellness Points | 7 | 7 |
| Wellness Points Added per Second with Minimum Stock of Health to Add Wellness Points | 0.12 | 0.12 |
| Minimum Stock of Health to Not Lose Wellness Points | 4 | 4 |
| Wellness Points Lost per Second if Stock of Health is Less Than Minimum Stock of Health to Lose Wellness Points | 0.14 | 0.14 |
| Health Stock Lost per Second of Walking | 0.11 | 0.11 |
| Units of Food Added per Second from Foraging Grass | 0.1 | 0.3 |
| Total Amount of Fruit on Trees | 120 | 40 |

Materials and Methods

Recruitment of experimental participants: The lab manager of the Economic Science Institute at Chapman University recruited the 96 undergraduate participants via email. All agreed to participate in the experiment before they read the specific instructions for this experiment. The Institutional Review Board at Chapman University approved the study.

Procedures: An author (Wilson) escorted the participants (50% women) into a computer laboratory in groups of 12 (6 women and 6 men) and randomly seated them at a visually isolated carrel. Each student only participated in one session. The participants read the computerized instructions at their own pace and practiced moving around the virtual world and performing all tasks. The experiment lasted for 52.5 min (35 days of 90 s each). At the conclusion of the experiment, the lab manager individually called each student to the window to privately pay them their earnings in the experiment (mean = US$16.13, s.d. = US$8.58, maximum = US$27.87, minimum = US$2.85), plus US$7 for showing up on time.

Instructions:

<page 1> **Welcome**

This is an experiment in the economics of decision making. The instructions are simple, and if you follow them carefully and make good decisions you can earn a considerable amount of money which will be paid to you in CASH at the end of the experiment.

In this experiment, you will be represented by the numbered avatar on your screen. You can move around the environment by *left* clicking
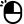
 on the spot you wish to move to. Notice that a red circle
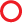
 marks the spot your avatar is moving towards.

<page 2> **Wellness and Your Earnings**

Each second your current level of wellness is
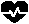
 converted into cash and added to your total cash earnings. The higher your wellness, the faster you earn cash.

During the **day**, your wellness will increase *or* decrease depending upon how much food
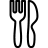
you have collected and how healthy **
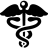
** you are.

At **night**
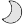
, your wellness will increase when you are sleeping, which will be explained later.

<page 3> **Food**
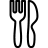


The bar in the upper right corner of your screen indicates how the food you have collected currently increases or decreases your wellness
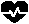
.

The bar will be displayed in green when you have a lot food, which will increase your wellness by 0.12 points per second.

Your current stock of food will gradually decrease as you move around the world. Eventually, the bar may change to yellow at which point food no longer increases your wellness.

If you do not replenish your food, the bar may turn red, at which point your wellness will *decrease* by 0.14 points per second.

<page 4> **Food**
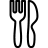


The timeline at the top of the screen indicates when your stock of food will rapidly drop (in the middle of the day and at the end of night).

Fruit
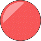
 grows on trees found throughout the environment. The map in upper left corner of your screen will show your location, denoted by the white box, as well as the location of other objects.

To replenish your stock of food, walk close to a tree and *right* click
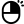
 on a piece of fruit, then *left* click
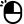
 the gather button. Fruit will increase your food by one point each time you harvest. A flower
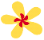
 will mature into fruit at the beginning of the next period. When a player consumes fruit, a fruit core
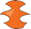
 will be left at their feet. An individual piece of fruit can be gathered and consumed only one time.

You can gather a piece of fruit, at most, once every 1 second(s).

Walk close to a tree, right click on a piece of fruit, and then press the gather button. Do this now.

<page 5> **Food**
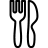


Grass
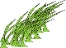
 can be found throughout the environment as well. Grass will increase your food by 0.10 [for *Chimpanzee* treatment and 0.30 for *Bonobo* treatment] each time you gather. In a given period grass can be gathered an unlimited number of times. At the end of the period all grass patches will die and regrow in a new location. Grass locations are shown on your map in the upper left corner of your screen.

To replenish your stock of food, walk close to a grass patch and grass will be automatically gathered.

It takes 10 second(s) [for *Chimpanzee* and 3.3 or *Bonobo*] to gather grass equivalent to *one* piece of fruit.

Walk close to a patch of grass to gather food. Do this now.

<page 6> **Health
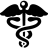
**

The bar in the upper right corner of your screen indicates how your health currently increases or decreases your wellness
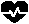
.

The bar will be displayed in green when you are quite healthy, which increases your wellness by 0.12 points per second.

Your current level of health gradually decreases as you move around the world. Eventually, the bar may change to yellow at which point your health no longer increases your wellness.

If you do not receive healing from another avatar, the bar may turn red, at which point your wellness will *decrease* by 0.14 points per second.

<page 7> **Health
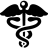
**

Another avatar must heal you for your health to increase. To heal another avatar, *right* click
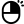
 on the avatar and press the heal button. You can only heal someone once every 4 second(s).

You cannot heal another avatar while you are eating grass.

Find and right click on an avatar now then left click the heal button. Do this now.

<page 8> **Calling**

The call
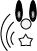
 button on the right side of your screen will alert other nearby avatars to your location. Similarly, if you see:


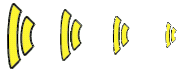


another avatar is calling you to their location.

A call will last for 10 seconds and turn off automatically. Other avatars are not visible on your map in the upper left corner of your screen.

Click the call button now.

<page 9> **Sleep, ZZZ …**

The sleep
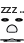
 button on the right side of your screen will put your avatar to sleep for the remainder of the period. When your avatar is asleep during the night
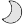
 your wellness
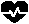
 will increase automatically by 0.30 every second. No actions can be taken at night even if your avatar is awake. The timeline at the top of your screen shows you when nighttime will occur. You cannot sleep during the daytime. A message will appear on your screen reminding you that night time is approaching.

Click the sleep button when next night approaches.

<page 10> **Pirates
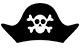
**

A pirate may appear above a tree and proceed to consume the fruit
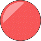
 on the tree. If a pirate is not stopped, it will consume all the fruit on a tree and return next period to a tree and consume all its fruit. To attack a pirate, walk near it then *right* click
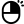
 on it then then *left* click
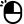
 the strike
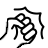
button. Striking a pirate takes 10 seconds and may hurt you. During which time you may not take any other actions other than calling
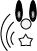
 other avatars to your location.

<page 11> **Conclusion**

There are help
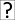
 icons on many of the objects in the experiment. Roll the mouse cursor over the icon and a help box will pop up. Many of the buttons will have a help box pop up if the mouse cursor hovers over them as well.

This is the end of the instructions. If you have any questions, please raise your hand and a monitor will come by to answer them. If you are finished with the instructions, please click the Start button. The instructions will remain on your screen until the experiment begins. We need everyone to click the Start button before we can begin the experiment. Your wellness will reset to **75.00** when the experiment begins.
